# Analgesic efficacy of intra-articular morphine after arthroscopic knee surgery in sport injury patients

**DOI:** 10.5249/jivr.v5i2.303

**Published:** 2013-06

**Authors:** Mitra Yari, Morteza Saeb, Parisa Golfam, Zahra Makhloogh

**Affiliations:** ^*a*^Clinical Research Development Center, Kermanshah University of Medical Sciences, Kermanshah, Iran.; ^*b*^Department of Anesthesia, Imam Reza Hospital,Kermanshah University of Medical Sciences, Kermanshah, Iran.; ^*c*^Department of Orthopaedic Surgery, Imam Reza Hospital, Kermanshah University of Medical Sciences, Kermanshah, Iran.; ^*d*^Student Research Committee, Kermanshah University of Medical Sciences, Kermanshah, Iran

**Keywords:** Intra-articular, Morphine, Arthroscopy, Knee

## Abstract

**Background:**

Anterior Cruciate Ligament (ACL) tearing is a common injury among football players. The present study aims to determine the best single-dose of intra-articular morphine for pain relief after arthroscopic knee surgery that, in addition to adequate and long-term analgesia, leads to fewer systemic side effects.

**Methods:**

This clinical trial was conducted on 40 ASA-I athletes. After surgery, all participants received an injection of 20cc of 0.5% intra-articular bupivacaine. In addition, the first control group received a saline injection and 5, 10 and 15 mg of morphine were respectively injected into the joints of the second, third and fourth groups by use of Arthroscopic equipment before the Arthroscopic removal. The amount of pain based on VAS at 1, 2, 4, 6 and 24 hours after surgery, duration of analgesia and the consumption of narcotic drugs were recorded.

**Results:**

The VAS scores in the fourth, sixth and twenty-fourth hours after surgery showed a significant difference between the study groups. The average time to the first analgesic request from the bupivacaine plus 15 mg morphine group was significantly longer than other groups and total analgesic requests were significantly lower than other groups. No drowsiness complications were observed in any of the groups in the first 24 hours after injection.

**Conclusions:**

Application of 15 mg intra-articular morphine after Arthroscopic knee surgery increases the analgesia level as well as its duration (IRCT138902172946N3).

## Introduction

One of the most common injuries among soccer players, especially in females, is tearing of the Anterior Cruciate Ligament (ACL) in the knee. This injury can be severely disabling and mean the end of an athlete’s career. Surgical repair is essential for stability and prevention of further injury. There are various surgical decisions that can influence outcomes.^1,2^

Previously the preferred surgical method for ACL tearing was primary repair , but because of recurrent instability in many patients, reconstruction of the ACL, usually with bone-tend on-bone (BTB) or hamstring grafts has replaced primary repair, and now this has resulted in objectively stable knees in 90% of patients and has become the gold standard for ACL treatment.^3^

To increase the level and duration of analgesia after arthroscopic joint surgery, non-steroidal anti-inflammatory drugs, and various kinds of narcotics such as sufentanil, tramadol and morphine have been used in addition to topical anesthetics.^4,5^In a study conducted in 2001 in England by P.N. Convery and et al. on 72 patients, different doses of intra-articular ropivacaine and bupivacaine were compared in terms of their concentrations in plasma. A 100 mg dose of intra-articular bupivacaine was reported as a safe dose for routine injection, because the concentration of created plasma is lower than the threshold of systemic toxicity.^6^

The effect of topical anesthetic can be prolonged in two ways: one is by increasing the drug’s amount, and the other is by adding a vasoconstrictor drug such as epinephrine or phenylephrine to the topical anesthetic or narcotic. The effect of the vasoconstrictor drug is to cause delay in the drug’s removal from the injection site and is short.

Peripheral administration of opioids (e.g. in knee arthroscopy) is associated with some clinical benefits. Because of its long duration of action, morphine is one of the narcotics that can be a good choice for this purpose. Morphine has an anti-inflammatory effect on joints and has no chondrotoxicity.^7^

In different studies, a relatively wide range of intra-articular morphine doses were used from 1mg to 15mg.^8,9,10^It is not clear whether adding a dose of morphine increases the level or duration of analgesia or not. On the other hand, it is not clear whether adding an intra-articular morphine dose increases systemic complications induced by opioids such as itching, nausea, vomiting and drowsiness or not.

The purpose of this study is to answer these two questions and to determine the most appropriate dose of morphine for intra-articular analgesia that, in addition to appropriate and long-term analgesia, will have fewer systemic side effects.

## Methods

**Figure 1:**
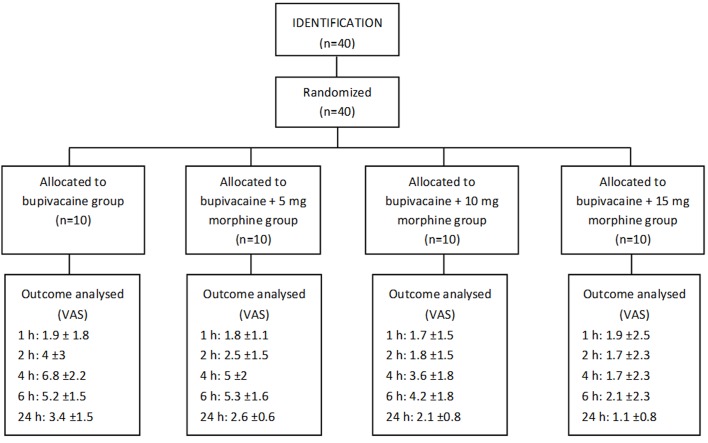
Patients progress through the trial: CONSORT Flowchart

This study was a double-blind clinical trial conducted on 40 ASA I athletes (healthy patients) aged 18-40 all of whom had undergone elective surgery for arthroscopic reconstruction of ACL. Patients who had a history of drug addiction were excluded from the study (Figure 1). Written consent was obtained from all participants. All patients received instructions for use of VAS (Visual Analog Scale) scaled from complete analgesia to the most severe imaginable pain and from 1 to 10. Anesthesia was induced with identical drugs such as 0.2 μg/kg Sufentanil, 3-5 mg/kg Sodium Thiopental and 0.5 mg/kg atracurium for facilitation of tracheal intubation. Anesthesia was continued with the maintenance of isoflurane and nitrous oxide. Patients who had a history of drug addiction were excluded from the study. Patients were randomly divided into 4 groups and at the end of trial, 20cc of 0.5% intra-articular bupivacaine plus 3cc intra-articular Normal Saline were injected before arthroscope removal using arthroscopy equipment in group 1. The same amount of bupivacaine plus 5, 10 and 15 mg morphine was injected into the joints of the second, third and fourth groups respectively. The volume of morphine was increased to 3cc by adding normal saline. The patient’s drain was also clamped half an hour after the injection. The amount of pain was recorded based on VAS criteria at 1, 2, 4, 6 and 24 hours after surgery. If the patient had pain, 10 mg intravenous tramadol was prescribed with every request of analgesia (Duration of analgesia was the interval between the completion of surgery and the first request of sedative). The amount of injected narcotic was also recorded after 24 hours. Itching, nausea, vomiting, and drowsiness were recorded at the listed hours to evaluate the systemic complications of narcotics. 

Patient information was recorded on two separate forms. In the first form, demographic data, type of intra-articular injected drug, the patient's name and case number were recorded. On the second form, the patient's name, hours since surgery completion and time of first request of analgesic and also possible complications were recorded. Intra-articular injection was done by the surgeon who was not then involved in the next monitoring and evaluation and data recording; furthermore, those completing the checklists and evaluating the symptoms were not aware of which groups the patients belonged to. Finally, data were statistically analyzed.

Nonparametric Kruskal-Wallis test was used to compare analgesia level in the four groups. If possible, ANOVA test and Tukey post hoc test were used to compare analgesia duration; otherwise the above-mentioned test was used. Chi-square test was used for possible complications. The SPSS software (version 13) was used for data analysis.

## Results

In total 40 people participated in this study. Ten patients were placed in each group. The average age of patients in these four groups was not significantly different (Table 1).

**Table 1:** Patients, age in study groups

Groups	Age (year)	p value
Bupivacaine	26.6±8	0.946
Bupivacaine + 5 mg morphine	28.6±15	
Bupivacaine + 10 mg morphine	28.4±8	
Bupivacaine + 15 mg morphine	26.3±6	

As shown in Table 2, in conducted evaluations the mean VAS score in the first and second hours after surgery showed no significant differences in the four groups studied. However, the mean VAS score in the fourth, sixth and twenty-fourth hours after surgery showed significant differences between study groups. This difference in the fourth and twenty-fourth hours was between the bupivacaine +10 mg morphine and bupivacaine +15 mg morphine groups and bupivacaine group. Moreover, there was a significant difference between the bupivacaine+ 15 mg morphine group and bupivacaine+ 5 mg morphine group. The only observed difference in the sixth hour after surgery was between bupivacaine +15 mg morphine and bupivacaine and bupivacaine +5 mg morphine groups.

**Table 2:** The results of intervention in study groups

		Bupivacaine	Bupivacaine + 5 mg morphine	Bupivacaine + 10 mg morphine	Bupivacaine + 15 mg morphine	p value
Postoperative pain (VAS)	1h	1.9 ± 1.8	1.8 ± 1.1	1.7 ± 1.5	1.9 ± 2.5	0.681
	2h	4 ± 3	2.5 ± 1.5	1.8 ± 1.5	1.7 ± 2.3	0.091
	4h	6.8 ± 2.2	5 ± 2	3.6 ± 1.8	1.7 ± 2.3	<0.0001
	6h	5.2 ± 1.5	5.3 ± 1.6	4.2 ± 1.8	2.1 ± 2.3	0.002
	24h	3.4 ± 1.5	2.6 ± 0.6	2.1 ± 0.8	1.1 ± 0.8	<0.0001
First narcotic request (hour)		2.8 ± 0.9	6.1 ± 1.6	8.9 ± 2.5	20.4 ± 6.2	<0.0001
Frequency of narcotic request (n)		2.6 ± 0.5	1.8 ± 0.9	0.9 ± 0.5	0.4 ± 0.9	<0.0001

Average time to the first analgesic request in patients receiving bupivacaine+15 mg morphine was significantly higher than other groups and no significant differences were observed between other groups. Also, the average number of analgesic requests in patients receiving bupivacaine +15 mg morphine was significantly less than the bupivacaine and bupivacaine +5 mg morphine groups. 

No drowsiness complications were observed in any of the four groups in the first 24 hours after injection. Four study groups showed no significant differences in terms of nausea and itching complications in the first 24 hours after injection, and both nausea and itching complications were observed only in one case and in the group receiving 5 mg morphine and in the second hour after injection. 

## Discussion

The study results showed that the prescription of bupivacaine and 15 mg morphine in comparison to 10 mg and 5mg morphine and bupivacaine has very beneficial effects on reducing the pain from arthroscopy surgery. The desired effects of a bupivacaine regimen with 15 mg morphine were remarkable. The lower pain score and number of analgesic requests in patients in the bupivacaine+ 15 mg morphine group reflected the appropriate pain relief produced by this regimen of drugs.

The mean VAS score in the first and second hours after surgery showed no significant differences in the four groups studied. Because bupivacaine is effective in relieving postoperative pain but its duration is usually short, patients need supplementary analgesia for subsequent hours. 

Morphine is likely to affect the μ receptor primarily and directly, but this effect may release endogenous opioids, which will influence Delta and Kappa receptors in an auxiliary way. Stimulation of peripheral μ receptors decreases sensory neuron activity and releases neurotransmitters in the present study, despite using a relatively high dose of morphine, we observed no complication. Poor lipid solubility of morphine hampers its passage across the synovial membrane into the blood stream and increases duration of the drug and the absorption of the drug into circulation is very low.^11^

In a study conducted in 2000 in Sweden by Brandsson et al. on 40 knee arthroscopy patients in 4 groups of 10 persons, 1 mg and 10 mg doses of intra-articular morphine were injected, serum concentration of morphine and its metabolite, morphine glucoronate, was measured and compared with serum concentration of the same doses after intravenous injection of morphine. Results showed that the systemic absorption of the injected drug is very low in the joint and the effect of analgesics and narcotics on the joint is due to the existence of peripheral mechanism in the joint, which reduces the pain without significant systemic effect.^8^ This result is similar to ours.

In different studies, a relatively wide range of intra-articular morphine doses were used from 1mg to 15mg. In 2007, Musil et al. in the Czech Republic compared the injection effect of the mixture of 10 mg morphine, 100 mg bupivacaine and 1 mg intra-articular adrenaline in arthroscopic knee surgery with a control group of 85 patients. The results showed the systemic reduction of a narcotic dose necessary for reducing pain after knee arthroscopy.^9^ This result supports the findings of this study to a significant extent. 

Some authors did not report dose dependant manner in morphine effects, some of them studied a lower dose of morphine. In 1999, McDermot et al. investigated the effect of adding 3 mg morphine to 50 mg intra-articular bupivacaine on 44 patients in England. The results showed that adding morphine does not significantly increase the analgesia level after surgery.^12^ Stein et al(1991) reported achieving effective analgesia with a small dose of morphine (1mg). However they noted delay in onset of analgesia and reported short duration(6h).

Different results were reported by Drosos et al. (2002) who compared 5 mg and 15 mg doses of intra-articular morphine with a placebo in 30 patients in three groups of 10 persons undergoing arthroscopic knee surgery. They concluded that the analgesia level is not significantly different between the two groups and the analgesia level is not increased after surgery with adding morphine dose from 5 mg.^10^ The difference might be explained by the fact that we have also used a local anesthetic drug with an early onset effect. It seems that bupivacaine with its pre-emptive analgesia mechanism causes an analgesia effect and using morphine often leads to a delayed effect. 

One of the most important elements related to the severity of pain after arthroscopy is the type of surgery, type of knee disorder, dose and volume and kinds of analgesic used and their combinations. Using tourniquet and timing of tourniquet release are also contributing factors in achieving special results.

In this study we used a relatively high dose of morphine in combination with bupivacaine. All of our patients were young and healthy and admitted for ACL insufficiency. 

Using intra-articular combination of 15mg morphine with 100 mg bupivacaine provides safe and long lasting analgesia after arthroscopy. In our study this combination was safe and free of side effects.

## Conclusion

The study results showed that adding intra-articular morphine to bupivacaine after arthroscopic knee surgery increases the analgesia level and duration. In this study, 15 mg of intra-articular morphine was compared to lower doses and was found to decrease systemic narcotics use and prolong analgesia duration without adding any complication such as itching, nausea, vomiting, and drowsiness. In summary our result showed that an increasing dose of intra-articular morphine provides better analgesic effect and showed linearly decreasing in VAS score and less supplementary analgesics.
